# Properties of Chitin and Its Regenerated Hydrogels from the Insect *Zophobas morio* Fed Citrus Biomass or Polystyrene

**DOI:** 10.3390/gels10070433

**Published:** 2024-06-29

**Authors:** Guillermo Ignacio Guangorena Zarzosa, Takaomi Kobayashi

**Affiliations:** Department of Science of Technology Innovation, Nagaoka University of Technology, 1603-1 Kamitomioka, Nagaoka 940-2188, Niigata, Japan; guillermo.guangorenaz@gmail.com

**Keywords:** insect, polystyrene, chitin, hydrogel

## Abstract

The potential of insects as a recycling tool has recently attracted attention. In this study, chitin was extracted with 1 M HCl for 24 h at 20 °C, followed by 1 M NaOH for 5 h at 90 °C, and bleached with 2.5% *v*/*v* NaOCl for 2 h at 20 °C from *Zophobas morio* (ZM) insects fed citrus waste biomass (OP) or polystyrene foam (PS). The highest survival rate was found in the OP group. The properties of the resulting chitin material are reported, as well as the preparation of hydrogels using a DMAc/LiCl solvent. All chitins obtained were α-chitin. The degrees of deacetylation, crystallinity, molecular weight, and solubility in DMAc/LiCl were similar between the PS and biomass feeds, and they showed similar viscosities in the DMAc/LiCl solution. All hydrogels obtained had similar properties and viscoelastic behavior, indicating that the resultant chitins and their hydrogels from ZM were similar between those fed with citrus biomass and those fed with PS.

## 1. Introduction

Chitin is a biopolymer composed of a linear homopolymer of ß-(1-4)-linked N-Acetyl-D-glucosamine. It is the second most abundant polymer in the world and is present in marine arthropods, mushrooms, and insects [1]. Approximately 100 billion tons of chitin are produced annually worldwide, mostly from marine arthropods, such as shrimps, crabs, and prawns [2,3]. It has an estimated market of USD 10.88 billion with growth expectations, particularly in Japan and the United States [4,5]. Recent studies have shown that insect chitin can produce higher yields than marine arthropods, such as cicadas [6,7]. Therefore, insects are an interesting source of chitin for places where there is not a marine food industry. With the growing demand for protein-rich food alternatives for humans, livestock, and animals, the insect industry may, in the future, be able to re-use biomass from breweries, agricultural waste, and pasture waste for protein production [8,9]. For this reason, some under-researched waste, such as citrus biomass waste, of which there is estimated to be about 1 million tons [10], could be used to breed insects. Considering insects as a resource, in addition to their high protein content, they consume less water for their protein production; emit less CO_2_, methane, and ammonia than other protein sources, and have the advantage of contributing to the development of a zero-waste society when they feed on waste [11,12].

Moreover, certain insects have attracted more attention from researchers for their capacity to eat plastic. Species such as the mole caterpillar (*Galleria mellonella*); the breadfruit (*Plodia interpunctella*); larvae of pest moths (Lepidoptera: Pyralidae), such as Indian mealmoths (*Plodia interpunctella*); greater waxworm (*Galleria mellonella*); lesser waxworms (*Achroia grisella*); and some species of darkling beetles of Coleoptera: Tenebrionidae (*Zophobas atratus*, *Tenebrio molitor*, *Tenebrio obscurus*, *Tribolium castaneum*, and *Plesiophthalmus davis*) have been described to eat plastic [13]. On the other hand, in species of the darkling beetle family, plastic eating has been studied, particularly in *Zophobas morio* and *Tenebrio molitor*. These two species can be fed polystyrene (PS) [14,15,16], low-density polyethylene (LDPE) [17], polyethylene (PE) [17,18], polypropylene (PP) [19], and polyvinyl chloride (PVC) [20] due to their gut microbiota. But even though they are capable of eating PS, some changes in their proteins and lipids related to oxidative stress were described [21]. However, the effects of a plastic diet on chitin, particularly for *Zophobas morio*, have not yet been made clear, with no studies available for this species. In a study on *Tenebrio molitor* on a plastic diet, chitin was extracted, but it was not used for material preparation [22].

On the other hand, there has not been much research on using chitin from insect resources as a material. This is because the use of chitin as a material is limited by its solubility, and its insolubility is based on its inability to dissolve in common solvents due to its strong intra- and inter-molecular hydrogen bonds [23]. But N,Ndimethylacetamide (DMAc) with lithium chloride (LiCl) has been used to dissolve insect chitin and fabricate hydrogels by an inverse coagulation method that showed similar viscoelastic properties similar to those of its marine counterparts [7]. Our recent studies have reported that chitin can be prepared from this chitin solution [24]. Chitin hydrogels can provide a matrix, with applications in the fields of biomedical, food, agriculture, and water treatment [25,26,27,28,29]. In general, hydrogels are three-dimensional structured networks of crosslinked hydrophilic polymer matrices with an abundant water content and different characteristics, such as softness, toughness, biocompatibility, stretchability, and deformability [30]. Recently, insect chitin hydrogels have shown similar properties to marine arthropod chitin hydrogels extracted under similar chemical treatments [7]. There are few studies on chitin materials from insects grown on waste biomass and plastic as feed. It would be very interesting to study them because they can contribute to a sustainable society and also convert waste into functional materials, such as chitin hydrogels. 

The aim of the current study is to compare plastic and biomass citrus waste as feed for *Zophobas morio* and their effects on the extracted chitins and their hydrogels. To our knowledge, this is the first study to compare the effects of diet on the physicochemical properties of chitin and its hydrogels extracted from *Zophobas morio* using DMAc/LiCl as the solvent.

## 2. Results and Discussion

### 2.1. Effects of Diet on Zophobas morio Survival Rate

The survival rate in the larvae varied according to the diet type, as seen in Table 1. The highest survival rate after 1 month of breeding was found in the biomass citrus waste (OP) group, with 82%. This was followed by the control group and the polystyrene (XPS) group, with 74% and 48%, respectively. Other experiments that evaluated the plastic-eating capacity of *Zophobas morio* (ZM) used two different larvae samples bred in different places (China and America). For the American *Zophobas morio*, a pure polystyrene diet could not keep the larvae alive, and they needed wheat bran supplementation. At the same time, the Chinese larvae could survive with only a plastic diet. After the American *Zophobas morio* were fed a mixture of polystyrene and wheat bran, they showed the lowest survival rate of 69%, while the Chinese insects showed a survival rate of 96% [17]. Another study group also using Chinese larvae showed a similar survival rate for both the control and polystyrene diet groups, at 68% [15]. It seems that the previous breeding conditions and the origin of the larvae had some influence on the survival of the ZM on plastic diets, yet all studies showed that ZM are able to eat plastic. Our study showed lower survival rates than the previously mentioned studies using Chinese larvae. On the other hand, outside wheat bran and oatmeal, biomass waste has not been researched in depth for ZM. One research group fed ZM with vegetable waste and green garden waste, with survival rates for both groups at around 92% [8]. Still, ZM is under-researched in comparison to *Tenebrio molitor* (TM). Most of the studies using biomass waste as a feed alternative have focused on TM. A study conducted in Thailand analyzed five different insects, including TM, on different diets using brewery waste and potato peals. Brewery waste (labeled as high-protein and low-fat) resulted in a survival rate of 67% [31], whereas just potato peals with wheat bran resulted in a survival rate of 92% [32]. In this study, citrus biomass waste was used without supplementation (mixed with wheat bran) and resulted in the highest survival rate of 82% within the samples, showing similar behavior to the ZM fed potato peals from other research groups. Overall, citrus biomass waste shows potential as a feeding alternative for ZM.

### 2.2. Effects of Diet on Properties of Chitin Extracted from Zophobas morio

Chitin was extracted from all samples, including the control and citrus waste (OP)- and polystyrene (XPS)-fed ZM. Table 2 summarizes the yield (%), the deacetylation degree (DA), the crystallinity index (CI), and the molecular weight (Mw, Mn) of the extracted chitins. All samples showed similar yield values of around 8% regardless of diet, so the diet did not affect the chitin yield rate. A similar chitin extraction was carried out in Tenebrio molitor fed polystyrene with a 10% (*w*/*v*) NaOH solution at 80 °C for 24 h, followed by a 7% (*v*/*v*) HCl solution at 25 °C for 24 h, showing yield of 6.78 and 5.81% for the control and polystyrene groups, respectively, but the extracted chitin was not fully bleached [22]. The Zophobas morio in the current study showed higher yield rates of 8% for all diet groups after alkaline, acid, and bleaching treatments, in contrast to those reported in the Tenebrio molitor. Using the whole insect compared to the cuticles affected the yield rate. To date, there has been no research evaluating plastic and biomass diets and their effects on chitin extracted from Zophobas morio, but depending on the chemical treatment used for the extraction, the usual yields are from 3.90 to 11.2% [7,24,33,34,35].

**Figure 1:**
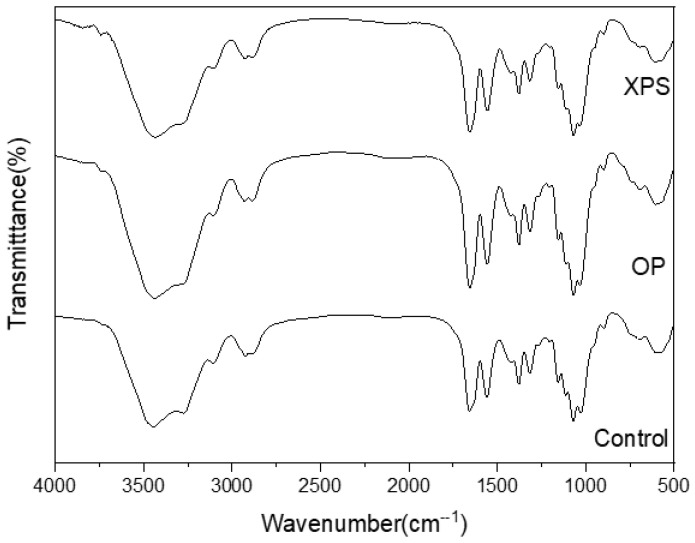
FTIR spectra of extracted chitins from different diet conditions.

The FTIR absorption spectra of the extracted chitins from ZM fed different diets are shown in Figure 1. In the spectrum data, the presence of stretching vibrations of the OH and NH_2_ vibrations corresponding to the acetamide were observed between 3450 and 3250 cm^−1^ [36]. Also, CH symmetric stretching can be seen at 2870 cm^−1^. A group of representative bands of chitin are those caused by amides I, II, and III, which appear around 1660, 1550, and 1370 cm^−1^. In amide I, the presence of two sharp bands around 1654–1652 cm^−1^ and 1619–1618 cm^−1^ can be observed. This splitting of the chitin amide I band is caused by the two types of hydrogen interactions that correspond to the intra- and inter-sheets in the antiparallel alignment present in the crystalline regions of α-chitin, which has also been reported in other insect-extracted chitins [6,35]. The bands corresponding to the symmetrical C-O-C stretching and C-O stretching of the pyranose ring are present at 1154, 1070, and 1058 cm^−1^ [37]. Similar bands have been reported in other ZM-extracted chitins [34,35]. The patterns of the spectral data of all extracted chitins are similar among the diets. A similar result was found in TM-extracted chitin when fed polystyrene [22]. In addition, the DA was also calculated and listed for every sample in Table 2. All extracted chitins showed DA values of around 69% regardless of diet. TM fed with polystyrene also did not show any differences from the control group-extracted chitin, with DA values of around 98% for both [22]. In this research work, the chitin was not bleached using oxidizing agents, such as sodium hypochlorite, which has an alkaline pH and can reduce DA values [7]. Differences in the concentrations and durations of deproteinization using sodium hydroxide have been described to alter the DA values of extracted chitins [34]. However, these differences can be attributed to the chemical treatment. But, similarly to the study conducted on TM, diet did not affect the DA values of the ZM-extracted chitin.

The elemental composition analysis of the extracted chitins was carried out using XRF, and the reports are listed in Table 3. Chloride was found in all the samples; this is attributed to the oxidizing bleaching agent, as seen in previous research work using sodium hypochlorite as a bleaching agent for chitin extraction from marine arthropods and insects [7]. The extracted chitins were similar among the samples; this suggests that the diets did not influence the elements found in the extracted chitins. Other works used EDS to determine the elemental composition, showing no differences in the TM-extracted chitins between the plastic and control diet groups [22].

**Figure 2:**
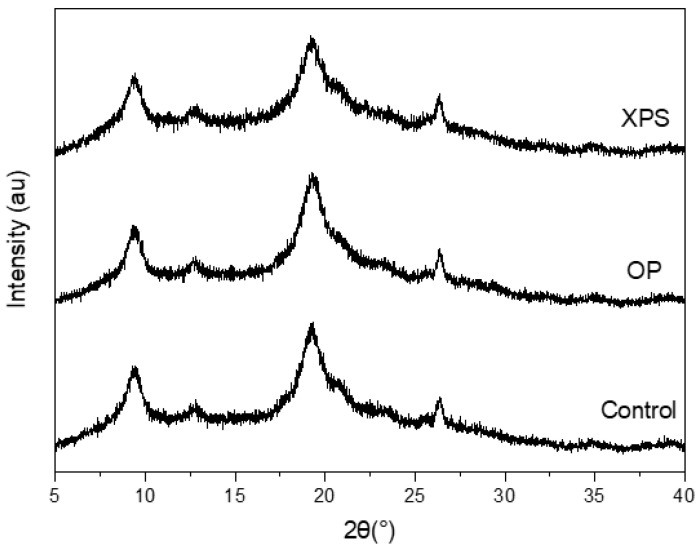
X-ray diffraction patterns of extracted chitins under different diet conditions.

Diffractograms obtained by X-ray diffraction (XRD) of the extracted chitins from all diet groups are presented in Figure 2. Their crystallinity index (CI) values obtained after the calculation are listed in Table 2. The diffraction patterns of the extracted chitins had characteristic peaks at 2θ of 9°, 19°, 23°, and 26° for the (020), (110), (130), and (013) planes, respectively, which are consistent with the α-chitin structure, especially the ones with two sharp peaks at 9.2 and 19.2, corresponding to the (020) and (110) diffraction planes, which can be observed in marine- and insect-extracted chitins [6,7,38,39,40]. The diffraction patterns and CI% values were similar between the TM fed plastic and the control group [22], similar to the results presented in this research using ZM. The CI% for all samples were around 62%, implying that diet did not affect the CI% of the ZM-extracted chitin. Other research works on ZM-extracted chitin have reported a CI% from 57 to 68%, depending on the chemical treatment [34,35].

**Figure 3:**
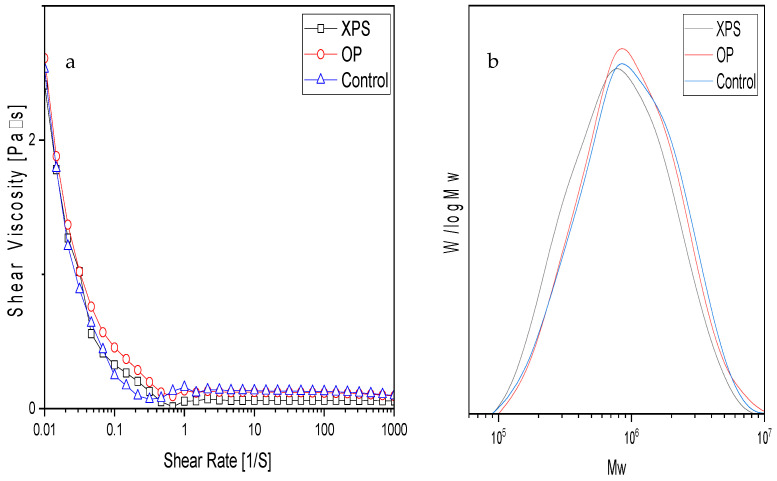
(**a**) Shear viscosity of 1% *w*/*w* DMAc/6% LiCl chitin solutions measured at 20 °C, and (**b**) molecular weight distributions.

Table 2 shows the molecular weight (MW) of all extracted chitins, the values of which were similar, at around 2.6 × 10^6^ g/mol. The GPC curves can be seen in Figure 3b. In a similar study conducted on TM-extracted chitin, the molecular weight of the chitin was not measured. Furthermore, other research groups that fed ZM polystyrene did not extract their chitin [15,22]. The results of this study suggest that the molecular weight of the extracted chitins was not affected by the diet. Differences in acid, alkaline, and bleaching during the chemical extraction of chitin have shown variations in the molecular weight of the extracted chitin products [7,34,41]. The solubility of all extracted chitins in DMAc/LiCl was nearly 100%. The viscosity of the 0.01% strains from all chitin solutions showed similar values, as seen in Figure 3a. The solubility values and viscosity are listed in Table 2. 

In addition, the viscosity graphs exhibit a shear-thinning flow behavior for all samples, where the viscosity decreases as the shear rate increases. At a resting state or nearly resting (0.01% strain), the polymer chains near each other are prone to entanglement. Once force is applied, the resistance to the flow decreases until it reaches a level high enough to disentangle the molecules to start flowing. The molecular weight of a polymer influences the viscosity at 0.01%, and the bigger the polymer chains, the higher the value at rest. This was seen for the OP chitin solution. Thus, the results suggest that food sources such as XPS or OP do not affect the molecular weight or solubility in DMAc/LiCl. Besides the chemical extraction, biological factors also interfere with chitin’s properties while it is being synthesized. In insects, the chitin is synthesized from ingested glucose that is transform in the fat tissue into trehalose. Trehalose travels through the endolymph to the cells responsible for chitin synthesis [42]. Furthermore, tyrosine is necessary for proper pigment synthesis [43,44]. Interfering with chitin synthesis alters the insect’s health. This is why some pesticides target chitins [42]. The homogeneity of the samples suggests that chitin metabolism is not affected by being fed XPS or OP. Meanwhile, changes in the proteins and lipids have been reported by other research works when polystyrene is fed to ZM. Chitin is a valuable option for those aiming to feed polystyrene to ZM. 

### 2.3. Effects of Diets on Chitin Hydrogel Properties from Zophobas morio

**Figure 4:**
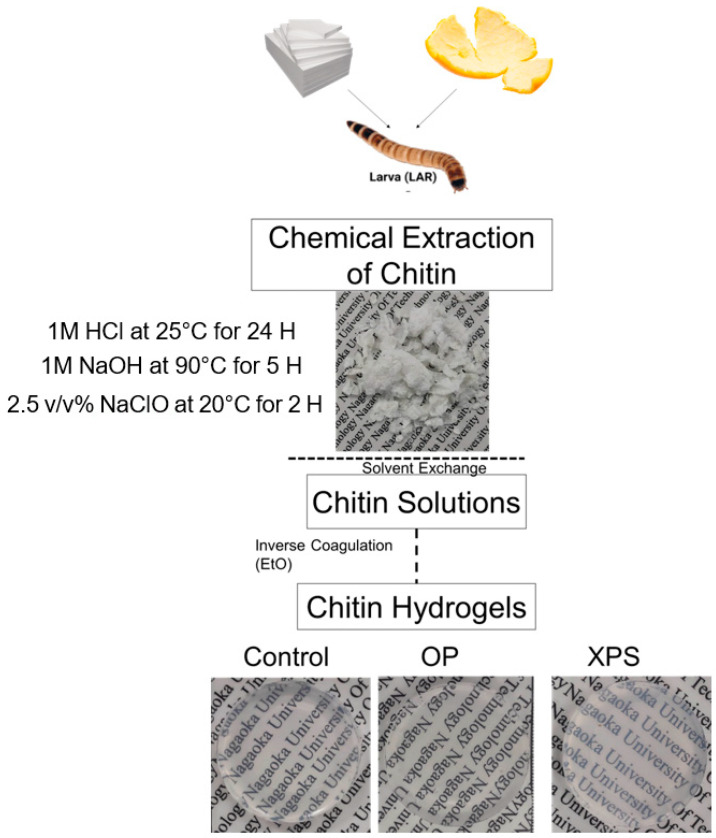
Chitin extraction and hydrogel preparation process.

After the phase inversion coagulation, hydrogels from all sample groups were successfully obtained from every dietary group. All chitin hydrogels were transparent, and their surfaces were regular, as shown in Figure 4. The hydrogels also showed similar values of water content, swelling rate, and contact angle, as listed in Table 4. All hydrogels showed hydrophilic behavior with high water content, around 15° and 97%, with similar swelling rates of 271%. The similarity among all samples suggests that the properties of the resultant gels were not different between the citrus biomass and plastic diets before the chemical extraction and hydrogelation process.

**Figure 5:**
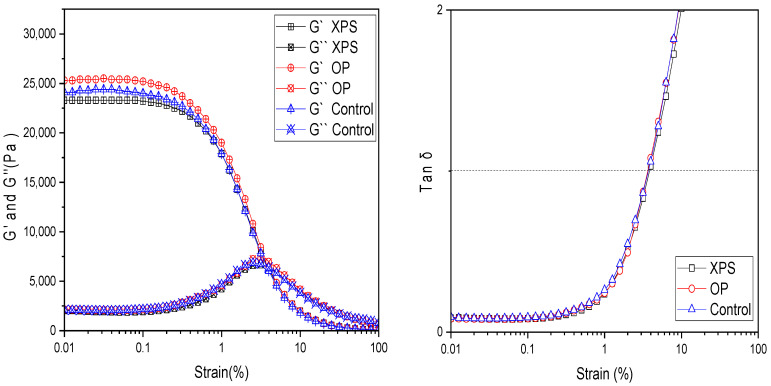
Strain sweep measurements of G′ and G″ and tan δ for chitin hydrogels measured from 0.01% strain to 100% strain at 20 °C.

Using a rheometer, the viscoelastic behavior was measured under mechanical strain (%) ranging from 0.01 to 100%. With this measurement, the storage modulus (G′) and loss modulus (G″) were plotted against the strain (%), as shown in Figure 5, to observe the mechanical deformation of the hydrogels under stress. For all chitin hydrogel samples, the G′ values were almost constant at strain values from 0.01 to 0.1%; this was also observed for the G″. But changes became evident after 0.1% strain; the G′ values of all hydrogel films started to decrease at a fast pace between 0.2% and 3.9% strain, suggesting that the hydrogels began to break under mechanical deformation. Similarly to G′, the changes in G″ were visible between the 0.2% and 3.9% strain values, and the loss modulus started to increase at a fast pace, indicating the loss of deformation energy due to the damage in the hydrogel structure. Yet, the samples predominantly display properties of a solid structure resisting the flow state. However, at around 3.9% strain, the transition point where G′ = G″ at tan *δ* = 1 was observed. The similarities of the swelling rates and tan *δ* = 1 values suggest that the crosslinking degree of the fabricated chitin hydrogels was similar among the samples. After this point, the viscous behavior dominated, suggesting that the hydrogel structure was broken and started to flow due to the applied strain. All samples showed a similar behavior as previously described, as shown in Figure 5. At 0.01% strain, the storage modulus (G′) values were 25,300 Pa, 24,100 Pa, and 23,300 Pa for the OP, control, and XPS groups, respectively. The slightly higher value for the OP can be attributed to its slightly higher molecular weight than the other samples. Overall, the samples were similar. This may be attributed to all extracted chitins showing similar values in DA, CI, MW, and solubility in the DMAc/LiCl solvent, leading to hydrogels with similar viscoelastic behaviors regardless of the food given to the larvae prior to the chitin extraction. Thus, the plastic diet or citrus biomass waste diet did not affect the properties of the hydrogels.

## 3. Conclusions

The insects fed the citrus biowaste mass (OP) diet showed the highest survival rate in this study. Chitin samples were obtained from the cuticles of *Zophobas Morio* fed citrus waste (OP), polystyrene (XPS), and oatmeal (control) diets. The chitin yield, and properties, such as deacetylation degree, crystallinity, and molecular weight were similar and comparable to those of other insect-extracted chitins. In addition, the extracted chitins, regardless of diet, showed similar solubility in DMAc/LiCl and viscosity values. Chitin hydrogels were successfully obtained, and they showed similar diameter, water content, swelling rate, and contact angle values, and similar viscoelastic behavior. This study shows the potential of ZM as a recycling tool for biomass and plastic, with the objective of chitin extraction for hydrogel preparation. Further research work is planned for insect chitin hydrogel applications in the biomedical field. 

## 4. Materials and Methods

### 4.1. Materials and Reagents

The larvae were purchased from a local breeder (Japan Fishing, Tokyo, Japan). Oatmeal (100 gr: 364 kcal, 14.2 g of protein, 6.9 gr of fat, 67.4 g of carbohydrates, and 12.3 g of fiber) and potato were purchased from a local grocery store (Nagaoka, Niiagata, Japan). Expanded polystyrene foam (EPS Board A3) was purchased from a local store (Daiso, Nagaoka, Niigata, Japan), and citrus waste was provided by the university as waste (Nagaoka, Japan). Sodium hydroxide (NaOH), N,N dimethyl acetamide (DMAc), lithium chloride (LiCl), sodium hypochlorite (NaOCl), and potassium bromide (KBr) were purchased from Nacalai tesque, Inc., Tokyo, Japan. Hydrochloric acid was purchased from Wako-Fujifilm, Tokyo Japan.

### 4.2. Insect Breeding and Survival Rate (%)

The XPS was cut into pieces of 2 × 2 cm^2^. The citrus waste was dried in 60 °C for 24 h and then blended into a powder. The oatmeal was blended into a power, and the lettuce was sliced prior to use. The *Zophobas morio* larvae were divided in three dietary groups, composed of 100 larvae per experimental condition, in triplicate, as follows: (1) the control group was fed 200 g of oatmeal, (2) the XPS groups was fed 20 g of expanded polystyrene foam (XPS), and (3) the OP group was fed 200 g of citrus waste dry powder (OP). Each experimental group was supplemented with 30 g of lettuce two days a week as a water source. The larvae were reared under 26 ± 1 °C and 30 ± 5% humidity on 20 × 15 cm 5L metal containers. The breeding was carried out for 30 days and repeated until a sufficient number of samples was collected for the extraction of chitin. For the survival rate (%), every week, the dead larvae were removed and the survival rate was recorded. For the survival rate (%) the following formula was used, where the initial larva (LI), and the measured larva (LM) were measured:

$$SR\left(\%\right)=100-(\frac{LI-LM}{LI}\times 100)$$

### 4.3. Extraction of Chitins and Hydrogel Preparation

For the chitin extraction, the control, OP, and XPS groups were used source materials. Figure 5 shows the chitin extraction process and the hydrogel preparation for each diet group. The chitin was extracted using the method shown in our previous report [24]. The *Zophobas Morio* was euthanized by freezing. The larvae were boiled at 80 °C for 15 min. Afterward, the meat was removed by hand, leaving the cuticles, which were used for the extraction of the chitin. For every dietary group, the cuticles were chemically treated and bleached as follows. The cuticles (30 g) were broken by hand, and then added to 900 mL of 1.0 M HCl and stirred at 20 °C for 24 h. Before the next step, the samples were washed with distilled water until pH = 7. The acid-hydrolyzed samples were then immersed in 900 mL of 1.0 M NaOH at 90 °C with stirring for 5 h to remove the proteins. Then, they were rinsed with distilled water, which was repeated until pH = 7. After the chemical extraction, the extracted chitin was dried in a vacuum oven at 60 °C for 24 h. The last treatment step consisted of submerging the extracted chitin (1 g) in 100 mL of 2.5 *v*/*v*% sodium hypochlorite at 200 rpm, while stirring for 2 h at 20 °C to remove the color. The bleached products were then collected by a mesh sieve and washed in 100 mL of distilled water at 200 rpm for 24 h. The distilled water was replaced every 2 h. Finally, the samples were dried in a vacuum oven at 60 °C for 24 h prior to their dissolution. For the preparation of the chitin solutions, the chitin (1 g) was suspended in 300 mL of distilled water, 300 mL of ethanol, and 300 mL of DMAc for the solvent exchange step. Each step was kept under constant stirring for one day. After the solvent exchange, 1 g of the chitin was suspended in DMAc/6% LiCl and stirred until most of the fibers were dissolved. To remove the insoluble parts, the chitin solutions were centrifuged at 30,000 rpm for 30 min. After the chitin solution was obtained, the hydrogels were prepared. Then, 10 g of the chitin solution was poured onto glass trays (5 cm diameter). The glass trays were placed in a sealed container and filled with 20 mL of ethanol at room temperature for 12 h. The ethanol vapor was generated, and its permeation with the DMAC/LiCl solution caused the formation of the chitin. After the hydrogels were fabricated, the films were autoclaved at 121 °C for 15 min and washed 3 times with distilled water for the removal of the DMAc and LiCl.

### 4.4. Characterization of Extracted Chitin

After the chemical extraction, the yields of the products were calculated. Using a FTIR spectrometer (Jasco 4100, Tokyo, Japan), with at a resolution of 4 cm^−1^ at 100 scans, the infrared spectra of each sample were captured. Then, using the spectra, the degree of N-acetylation (DA) was calculated using the following formula:

$$DA\left(\%\right)=\frac{1}{1.33}\left(\frac{A1655}{A3450}\right)\times 100$$

where A1625 and A3450 are absorbances measured at 1625 and 3450 cm^−1^, respectively.

Using an X-ray diffractometer (XRD, Rigaku Smart Lab 3 kW, Tokyo, Japan) under operating conditions of 40 kV and 30 mA with Cu–Kα radiation, the diffractograms were obtained. The relative intensity was recorded in steps of 0.01° and a speed of 2.0°/min. Using the diffractogram data, the degree of crystallinity (CrI) was determined using the following formula:

$$CrI\left(\%\right)=\left(\frac{I110-Iam}{I110}\right)\times 100$$

where I_110_ denotes the maximum intensity of the I_110_ crystalline peak at plane 19°, and I_am_ denotes the minimum intensity of amorphous diffraction at 16°.

### 4.5. Characterization of Chitin Solutions

The chitin solubility in DMAc/LiCl was calculated by collecting the insoluble chitins after centrifugation, followed by washing with distilled water to remove remnants of the solvent used. After being washed, the insoluble chitin was dried in an oven at 60 °C for 24 h, and its weight was measured. The following formula was used to calculate the solubility, where C1 is the chitin used to prepare the chitin solution, and C2 is the recovered chitin after centrifugation:

$$Chitin\;Solubility\left(\%\right)=\left(\frac{C1-C2}{C1}\right)\times 100$$

Using a Rheometer (Physica MCR 301, Anton Paar, Graz, Austria), the viscosities of the chitin solutions were recorded at a shear rate of 0.01 to 1000 s^−1^ at 20 °C.

Using a size exclusion chromatography equipped with a refractive index detector (RID-10A, Shimadzu, Kyoto, Japan) and a chromatographic column (KD-806 M, Shodex, New York, NY, USA) at 60 °C, the molecular weights of the chitins (after bleaching) were measured. The peaks of the chitin solutions were compared with those of polystyrene standards (Tosoh Corporation, Tokyo, Japan), and then these data were used for the estimation of the extracted chitin molecular weight. 

### 4.6. Characterization of Chitin Hydrogels

The hydrogel films with a thickness of 5 mm were cut into 2 cm^2^ diameter circles. The films were immersed in excess water for one day. After this, the water content was measured. The wet films were recorded (W1). The films were then dried in a vacuum oven for 24 h at 60 °C, and then weighed (W2). Finally, the following formula was used:

$$Water\;Content\left(\%\right)=\left(\frac{W1-W2}{W1}\right)\times 100$$

Using Auto Paar-Reoplus equipment (Physica MCR 301, Anton Paar, Graz, Austria), the viscoelastic behavior of the wet hydrogel films was measured. The storage modulus (G′) and loss modulus (G″) were measured from a 0.01% to 100% strain range at a 1 Hz frequency at 20 °C using a parallel plate head. The values of tan = G″/G′ at each strain rate were also recorded.

Using a contact angle goniometer (Kyowa Interface Science, Tokyo, Japan), the contact angle was measured for the fabricated chitin hydrogels. Pieces of 2 × 2 cm were cut for each sample and then mounted on a glass slide to drop a total of 3 µL of double distilled water on the air-side surface of the films at room temperature. The measurement was made 10s after the droplet touched the film surface, and this was repeated at least 8 times to obtain the average result.

To calculate the swelling ratio, the hydrogels were freeze-dried, and their weights were recorded as (Wa). Subsequently, the samples were immersed in distilled water until equilibrium was reached, and the weights of the swollen samples were recorded as (Wb). The swelling ratio was determined using the following formula:

$$Swelling\;Ratio\left(\%\right)=\left(\frac{Wb-Wa}{Wa}\right)\times 100$$

## Figures and Tables

**Table 1:** Weekly survival rate (%) of *Zophobas morio* on different diets.

Feeding Group	Week 1	Week 2	Week 3	Week 4
Control	89.6 ± 2.5	85.6 ± 5.0	80.6 ± 3.0	74.6 ± 4.5
OP	99.5 ± 0.7	97.5 ± 0.7	90 ± 2.8	82.5 ± 0.9
XPS	84.6 ± 6.1	74.3 ± 11.8	59.3 ± 3.3	48.6 ± 8.5

**Table 2:** Summary of yield, deacetylation degree (DA), crystallinity index (CrI), solubility, molecular weight of chitins, hydrogel water contents, and contact angle.

Feeding Group	Yield (%)	DA (%)	CrI (%)	Mw (10^6^ g/mol)	Mn (10^6^ g/mol)	Viscosity * (Pa·s)	Solubility ^+^
Control	8.4 ± 0.6	69.6 ± 0.7	62.9 ± 1.5	2.5 ± 0.02	2.0 ± 0.01	2.5 ± 0.7	96.4 ± 1.5
OP	8.5 ± 0.8	69.2 ± 1.6	62.5 ± 0.7	2.6 ± 0.04	2.1 ± 0.07	2.6 ± 1.2	96.2 ± 1.1
XPS	8.4 ± 0.7	69.5 ± 1.9	61.9 ± 1.2	2.5 ± 0.03	2.0 ± 0.04	2.4 ± 1.7	95.8 ± 1.7

* Viscosity at 0.01% strain of chitin in 1% *w*/*w* DMAc/6% LiCl; ^+^ solubility in DMAc/6%LiCl.

**Table 3:** Elemental composition of each extracted chitin.

Sample								Elements							
	C	N	O	Na	Mg	Al	Si	P	S	Cl	K	Ca	Mn	Fe	Zn
Control	44.3	8.07	45.5	0.0325	0.0943	-	0.0456	0.003	-	1.92	0.005	0.066	-	0.0118	-
OP	44.5	8.01	45.6	0.0401	0.0890	-	0.0459	0.007	-	1.42	0.005	0.068	-	0.0152	-
XPS	43.8	8.09	46.6	0.0311	0.0903	-	0.0545	0.007	-	1.63	0.008	0.036	-	0.0120	-

**Table 4:** Chitin hydrogel water content (%), contact angle (°), and swelling rate (%).

Chitin Hydrogel	Water Content (%)	Contact Angle (°)	Swelling Rate (%)
Control	97.7 ± 0.7	15 ± 1	271.3 ± 0.5
OP	97.6 ± 0.2	14 ± 1	271.5 ± 0.4
XPS	97.8 ± 0.5	15 ± 1	271.3 ± 0.4

## Data Availability

The data that support the findings of the current study are listed within the article.
